# Occult Hepatitis B (OBH) in Clinical Settings

**DOI:** 10.5812/hepatmon.6126

**Published:** 2012-08-25

**Authors:** Seyed Moayed Alavian, Seyed Mohammad Miri, F. Blaine Hollinger, Seyed Mohammad Jazayeri

**Affiliations:** 1 Baqiyatallah Research Center for Gastroenterology and Liver Disease, Baqiyatallah University of Medical Sciences, Tehran, IR Iran; 2 Baylor College of Medicine, One Baylor Plaza, Houston, TX, USA; 3 Department of Virology, School of Public Health, Tehran University of Medical Sciences, Tehran, IR Iran

**Keywords:** Hepatitis B, Hepatitis B Surface Antigens, Coinfection

## Abstract

**Context:**

Occult hepatitis B (OHB), or persistent HBV DNA in patients who are hepatitis B surface antigen (HBsAg) negative, is a recently recognized entity. In an attempt to summarize the issues, this review presents an overview of the current proposed hypothesis on the clinical relevance and also updates the knowledge on the classification of OHB in different clinical settings.

**Evidence Acquisition:**

OHB could be found in different population and clinical backgrounds including: viral co-infections (with either human immunodeficiency or hepatitis C viruses), HBV chronic carriers, dialysis patients, transplantation settings and certain clinical situations (named in here: special clinical settings) with no apparent distinguishable clinical parameters.

**Results:**

The exact magnitude, pathogenesis, and clinical relevance of OHB are unclear. Even the possible role exerted by this cryptic infection on liver disease outcome, and hepatocellular carcinoma development remains unknown.

**Conclusions:**

Monitoring of Individuals with positive anti-HBc, mass immunization programs and improvement in diagnostic tools seem to be important to control the probability of transmission of HBV through cryptic HBV infection.

## 1. Context

### 1.1. Occult Hepatitis B in Clinical Settings

Occult hepatitis BHBV infection has been documented in a variety of clinical situations, especially among patients who are positive for anti-HBc (Table 1). Occult HBV infection is also commonly present in HCC, chronic hepatitis C virus (HCV) infection (1, 2), human immunodeficiency virus (HIV) infection (3), liver-transplant recipients of a liver from a donor who was antibody to the hepatitis B core antigen (anti-HBc) from core-antibody-positive donors (4), hemodialysis patients (5), healthy carriers, patients with cryptogenic advanced liver fibrosis (6), and special high-risk patients.

**Table 1:** Reported Prevalence of OHBI Obtained From Blood Specimens Collected From Different Clinical Settings

**Clinical Setting**	**OHB a Prevalence, %**	**Prevalence of OHB in Anti-HBc a Positive Patients, %**
Blood donors b	0.05-13	0-17
HIV a	0-89	9-44
HCV a	6.7-91	28-71
HCC a,c	12-80	28.8-64
Immunosuppression	3.3-37.8	37.8-62.3
Dialysis	0-58	6.4-64.7
Chronic HBV carriers	5-55	7-60
Cryptogenic cirrhosis	4.8-40	17.8-100
Transplantation	36-64	3-100
Liver	0-50	4.4-100
Stem Cell	0-3.3	3-10
Kidney		
HBV a vaccinated	2.7-28	6.5-100
Family contact of HBsAg positive carriers	8.8- 28.8	23.6-96.4
General Healthy Population d	0.7-34	6.1-51
Hemophilia	5.3-51.2	6-100

^a^Abbreviations: HBc, hepatitis B core; HBV, hepatitis B virus; HCC, hepatocellular carcinoma; HCV, hepatitis C virus; HIV, human immunodeficiency virus; OHB, Occult hepatitis B.

^b^The lower and upper limits are in the presence of other serologic evidence and isolated- anti-HBc, respectively.

^c^No HCV-related HCC.

^d^Highly dependent on the geographic prevalence of HBV.

## 2. Evidence Acquisition

The primary aims of this review were to: first, classify the different aspects of occult hepatitis in terms of clinical classification. Second, to describe the prevalence of OBH in different clinical settings. Third, to introduce an up to date information regarding the clinical and transmission aspects of OBH. A comprehensive search of PubMed was performed with the following Mesh term search using keyword: occult hepatitis B infection along with keywords: HCV, HIV, Immunosuppression, HCC, transfusion and transplantation. All published data since 2001 (the explanation of OBH) until May 2012 have been included in the study. The inclusion criteria for the study were: all studies that shown the presence of HBV DNA in the absence of HBsAg regardless of antibodies to core and/or surface proteins.

## 3. Results

### 3.1. Transfusion Settings

The blood-safety issues related to the blood components or tissues of HBsAg-negative donors and the risk of transmission are of great concern. Medical researchers fear that OHBI may be spread by blood transfusion because HBV-DNAHBV DNA detection by nucleic acid amplification technology is not mandatory for blood-donor screening in many countries, especially in those areas where the prevalence of escape mutants is expected to be high. Occult HBV infection may occur in several situations and, as a result, may be transmitted to other individuals through transfusion. Such situations include recovery from infection that leads to the production of anti-HBs but is characterized by persistent low-grade viremia, production of ‘escape’ mutants that cannot be detected by HBsAg testing, “carriers” with antibody to the hepatitis B e antigen (anti-HBe) and anti-HBc, and carriers that are negative for anti-HBe and anti-HBc. Although all of these situations can raise the transmission of HBV in the immune compromised, only the anti-HBc-positive, HBV-DNA-positive situation is infectious in the immune competent setting. Furthermore, Studies have clearly indicated that transfusion of HBsAg-negative, HBV DNA positive blood donor in blood supply was associated with the development of post-transfusion hepatitis B (7, 8, 9). The prevalence of OHB among HBsAg-negative donors varies in different parts of the world, and as mentioned above, depends on the endemicity of HBsAg carriers as well as the method used to test the virus. Reports from subsets of donors from North America and Western Europe have ranged from as low as 0 % (presence of anti-HBc) (10, 11) to as high as 2.8 % (isolated anti-HBc) (12, 13). Similarly, studies from Asia (especially from highly endemic regions) have indicated prevalence rates of HBV-DNAHBV DNA detection among blood donors ranging from 0.13 (presence of anti-HBc) (14, 15) to 8% (isolated anti-HBc) (16) (Table 1). As in other viral infections, HBV infectivity depends on two main factors: the infectious dose and the immune competence of the host. Considering the volume of infectious material involved in the transfusion of whole blood or a blood component, it is generally accepted that should any HBV DNA be present, infection may occur (even with a viral load of < 20 IU/mL) (17). However, some scientists have argued that blood containing anti-HBc with anti-HBs does not appear to transmit HBV components (even through viral loads ranging between 20 and 500 IU/mL) (8, 17) if the neutralizing antibody level is sufficiently high.

### 3.2. Chronic Carriers

OHB in apparently healthy HBV chronic carriers is essentially found in four types of clinical conditions: 1) recovery from infection defined by the presence of anti-HBs (spontaneous HBsAg seroclearance in a carrier) (18); 2) chronic hepatitis, where the infection is related to escape mutants that are not (or are only poorly) recognized by either natural polyclonal or monoclonal antibodies in the assays (19); 3) low-replicative phase of chronicity at the healthy carriage stage marked by the presence of anti-HBc, with or without detectable antibody to hepatitis B e antigen (anti-HBe) (20); and 4) chronic hepatitis or healthy carriage without any marker of HBV infection other than HBV DNA (21, 22), this illustrates the fact that some patients may have had serological markers of HBV infection, but subsequently lost them while still continuing to have a low- grade HBV infection (22). OHB has been described in chronic HBV infection with different outcomes from asymptomatic carriers to HCC patients with genotypes A through F and H (23, 24, 25, 26, 27). Several authors have investigated HBV influence ,and different HBV genotypes on OHB prevalence in specific regions. For instance, Weinberger et al. (28) suggested an association between occult HBV infection and genotype D in individuals from Western Europe, where genotype A is more prevalent. However, the findings from a French study did not support this association (29). Cohort studies that include different HBV genotype patients are needed to compare OHB implications in these genetic groups of patients HBV.

### 3.3. HCV

Among 350 million HBV carriers worldwide, the number of individuals with HCV–HBV dual infection is around 5 – 7 million. However, it is very likely that the dual HBV–HCV infection prevalence rate is underestimated because occult HBV infection is often not taken into account (30). Since HBV and HCV share many risk factors and the same transmission routes, the high prevalence of occult HBV infection reported in patients with chronic hepatitis C, ranging from 6.7% to 91.1%, is not surprising (31, 32). It is well established in the medical literature that HCV-positive HCV positive patients exhibit the anti-HBc-alone pattern more often than do HCV-negative HCV negative patients, and anti-HBc reported prevalence in patients with chronic HCV infection ranges from 28 % to 71 % (33, 34, 35) (Table 1).

These differences in prevalence rates might be responsible for inconsistencies in results on specimen types, recruitment of chronic HCV-infected patients, HBV infection endemicity in the study areas, and study methods in general. One clear example comes from Sagnelly et al. (31) who found prevalence rates of OHB in HCV -infected patients as high as 91.1 % in liver samples compared to just 62.2 % and 32.4 % in PBMC and plasma samples, respectively. Regarding the clinical consequences of occult HBV infection in patients with chronic hepatitis C, studies have indicated the negative influence of cryptogenic HBV infection on histologic activity and severity of liver disease and HCC, possibly by integration with the host genome or synthesis of pro-oncogenic proteins by free intrahepatic HBV genomes (34, 36, 37, 38). Also, in HCV- infected patients, occult HBV infection may contribute to increased plasma HCV-RNA loads and liver transaminase levels (39, 40). On the other hand, some studies have reported that OHB occult HBV infection does not affect the pathological findings in the liver, changes in aminotransferase levels, or occurrence of HCC in patients with chronic HCV (32, 41, 42). It should be noted, however, that all of these studies are cross-sectional, and therefore prospective studies are needed to confirm that occult HBV accelerates liver lesions progression in patients with chronic hepatitis C. Occult HBV infection also might affect the response to antiviral therapy in patients with chronic hepatitis C (43, 44), but the available data do not allow any firm conclusions in this regard, partly because of the heterogeneity of the patients enrolled, and the lack of data on care current standards for treatment (45). One study (43) found that HCV patients with occult HBV had lower intrahepatic mRNA levels of IFNAR2 (one of the two subunits of the IFN receptor), which the authors argued was one of the factors that could have led to a poor IFN response. However, the mechanism by which occult HBV may inhibit the patient’s response to IFN therapy in chronic hepatitis C remains unknown and therefore merits further research. Regarding the interaction between different HCV genotypes and OHB, Fukuda et al. (43) observed that patients with occult HBV infection were more frequently infected by HCV genotype 1a, but this association was not observed in other studies (32, 44). Altogether, these data highlight the clinical implications of occult HBV infection in the clinical setting of HCV infection and suggest that screening for OHB should be an essential practice.

### 3.4. HIV

Due to transmission shared modes and risk factors, co-infection with HBV and HIV and even triple infection with HCIV is common. A high HBV DNA prevalence in HBsAg-negative samples from HIV-positive patients indicates that HIV infection is a risk factor for OHB (46). Indeed, many reports have indicated that HIV-infected patients are at a higher risk of HBV co-infection in many regions, as illustrated by HBsAg high prevalence in HIV-positive patients in comparison to HIV-negative patients (47, 48). The reported prevalence of OHB occult HBV among HIV-infected patients has ranged from 0 % to 89.5 %, mostly in anti-HBc positive patients, with a considerable number of other studies reporting results between these two extremes (49, 50, 51) (Table 1). The variability between the studies may be explained by fluctuations in HBV-DNAHBV DNA replication over time (even when these assays are validated with high specificity and quite high sensitivity), HCV co-infection, or the impact of treatments that are active against both HIV and HBV, such as lamivudine and tenofovir. In addition, differences in risk factors for contracting HBV between the studied populations and regional differences in overall HBV prevalence may contribute to the observed differences in occult HBV prevalence. Furthermore, such studies suffer from a lack of methodological standardization, limited sample sizes, and few prospective studies. Occult HBV infection clinical significance in HIV-infected patients remains unclear. HBV reactivation from occult to overt HBsAg infection can occur in immune suppression clinical setting (52), which underscores the potential clinical significance of occult HBV infection in HIV-positive persons. Apart from HCV infection influence on the prognosis of OHB and HIV, multiple studies have reported an association between occult HBV infection and flare-ups of hepatic transaminases (49, 53, 54). One study found that these liver outbreaks were due to the restoration of adaptive HBV-specific immune response and innate nonspecific immune responses (55), although several other studies that compared the levels of liver enzymes in HIV patients with and without OHB were not able to confirm these response routes (51, 56, 57, 58). Longitudinal studies with longer periods of observation that examine other hepatic outcomes, such as hepatic fibrosis by liver biopsy and hepatocellular carcinoma, would provide more insight into occult HBV clinical impact in HIV-infected individuals. The literature generally advocates that, for individuals co-infected with HBV and HIV, HBsAg- positive patients should be treated if their HBV DNA, or aminotransferase levels increase, or if they experience significant hepatic fibrosis (59). Still, clear guidelines have yet to be established for occult HBV co-infection in HIV positive patients.

### 3.5. Cryptogenic Cirrhosis

Several studies have also demonstrated the presence of HBV DNA in the serum or livers of patients with cryptogenic cirrhosis, who lack serologic markers for HBV and HCV (34, 60, 61, 62, 63). The frequency of occult HBV infection in cryptogenic cirrhosis has ranged between 4.8 % and 40 % in various studies, depending on the prevalence of HBV in the study areas and the type of specimen studied (serum or liver) (34, 60, 62, 64) (Table 1). OHB clinical features in these settings remain largely unknown. In the laboratory, no association was found between the presence of OHB and demographic, biochemical (AST, ALT), or serologic features in cryptogenic cirrhotic patients, implying that none of these parameters were useful to distinguish OHB-positive from OHB negative patients. However, observations showed that OHB frequency increased in anti-HBc positive patients isolates (regardless of anti-HBs positivity) (60, 61, 62, 64). Cirrhosis is generally considered as the most important risk factor for hepatocellular carcinoma development; therefore, in addition to its possible direct oncogenic properties, occult HBV infection may favor neoplastic transformation in infected patients through its contribution to cirrhosis.

### 3.6. Hepatocellular Carcinoma (HCC)

Despite the strong association between HCC and seropositivity of HBsAg, almost all clinical and epidemiological studies have observed HBsAg-seronegative patients affected by HCC and liver cirrhosis. A significant proportion of these patients were seropositive on anti-HBs and anti-HBc seropositive and seronegative foron antibodies against hepatitis C virus (anti-HCV), especially in areas where HBV infection was hyperendemic (35, 65, 66, 67, 68). Several studies have reported HBV DNA detection in tumorous liver tissue of HBsAg- negative HCC patients, with prevalence rates ranging from 30 % to 80 % (36, 69) (Table 1). The major question that arises with this evidence is whether occult HBV infection alone causes HCC. The hypothesis that OHB occult hepatitis retains the same pro-oncogenic features,accelerates liver disease progression, and cirrhosis development (36) has been suggested by epidemiological and molecular studies and supported by animal models. Accordingly, experiments in animal models demonstrated that both woodchucks and ground squirrels, once infected by woodchuck hepatitis virus and ground squirrel hepatitis virus, respectively, were at high risk of developing HCC after the apparent clearance of the virus (70, 71).

Three longitudinal follow-up studies showed the role of OHB in HCC development (65, 72, 73). In follow-up periods of 82.8 ± 32.6 months, eight out of nine patients who acquired HCC were OHB positive at the time of final follow-up (73). In a similar finding, in a period of 91.8 ± 48.5 months, four out of eight OHB positive cases had acquired HCC (72). However, one follow-up study found a negative correlation (74); specifically, only one of seven OHB and HCV co-infected patients whose HCV was not without HCV eradicated ion with interferon therapy developed HCC during average periods of 58.6 ± 18.1 months. Future cohort studies must be performed to address this question. Mutational analysis in HBsAg-positive HCC versus HBsAg-negative patients showed multiple genetic variants in different parts of the HBV genome and regulatory regions (68, 75, 76); however, others did not find such an association (41, 77). Furthermore, in a direct examination using cassette-ligation-mediated PCR in the junctions between HBV DNA and human DNA, the integration of HBV DNA was identified in 10 out of 34 HCC patients, all of whom were OHB positive. Four of these integrations were observed in chromosome 11q (78). Altogether, clear evidence exists that OHB infection HBV maintains its pro-oncogenic properties in case of occult HBV infection, possibly by integrating with the host genome, the synthesis of pro-oncogenic proteins by free intra-hepatic HBV genomes (33, 36, 47, 52, 64, 69), or by progression from through cirrhosis to hepatocellular carcinoma (73). However, to reach the conclusion that OHB plays a major role in hepatocellular transformation, additional studies on molecular pathogenesis and prospective molecular epidemiological studies are needed.

### 3.7. Immunosuppression

Hepatitis, due to reactivation of HBV, is now a well-recognized complication in patients with chronic HBV infection receiving cytotoxic, corticosteroid, or immunosuppressive therapy. In these cases reactivation of the virus may occasionally be present, leading to severe or even fulminant hepatic failure sometimes indistinguishable from de novo acute infection (34, 52, 79). Several reports have found that patients with hematological malignancies had a high carrier rate of overt HBV (i. e., HBsAg positivebetween23.8 and 56 %) (80, 81). Also, evidence of previous HBV infection (especially the presence of anti-HBc) varies among studies, rangingfrom37.8 % to 62.5 % (81, 82, 83) (Table 1). In this respect, HBV reactivation has been observed in two clinical settings: 1) advanced immune deficiency in patients with hematological-malignancy disorders, (such as acute leukemia, myeloproliferative disorders, lymphoproliferative disorders, and plasma cell dyscrasias,) at a prevalence of 3.3 % to 24 % (80, 81, 82, 83); and 2) subjects who were HBsAg negative prior to chemotherapy (alkylators, antimetabolites, antitumor antibiotics, corticosteroids, etc.) who underwent treatment and transplantation (bone marrow, liver, kidney, and hematopoietic stem-cell transplantation HSCT), especially those treated with the immune suppressive rituximab (anti-CD20), alemtuzumab (anti-CD52), and infliximab (anti-TNF) (82, 83, 84). The rate of reactivation after chemotherapy has been reported to range between 3.3 % and 4 % among OHB-positive immunosuppressed patients after treatment (82, 83). However, there are numerous case reports of HBsAg-negative patients suffering from HBV reactivation after treatment by rituximab (84, 85), alemtuzumab (86, 87), and infliximab (as a therapeutic regimen for rheumatic and Crohn’s diseases) (88, 89) , and the number of these studies is increasing. HBV reactivation following chemotherapy can resolve, persist, recur, or lead to liver failure and death. However, viral proliferation and progression of the disease increase when patients with HBV infection immune response is suppressed by immune suppressive or cytotoxic agents. On the other hand, withdrawal of these agents leads to the immune-mediated destruction of HBV-infected hepatocytes and hepatitis flares. Such flare-ups have been attributed to changes in equilibrium due to an enhanced immunological response to HBV during recovery from immune suppression (90, 91). In addition to the direct effects of immunosuppression, it has been indicated that the HBV genome contains a steroid-responsive DNA sequence that, when stimulated, results in an increase of HBsAg synthesis and HBV gene reactivation (92).

### 3.8. Transplantation

One of the most important clinical implications of OHB is usually observed in the setting of liver transplantation. In particular, livers from donors with OHB carry a risk of HBV transmission with infection occurring in 25–95 % of the liver grafts donated from patients who are HBsAg negative but anti-HBc positive. This infection route is indistinguishable from overt infection (9, 93, 94, 95) (Table 1). The significance of OHB in post-OLT (orthotropic liver transplantation) settings is controversial. Upon intrahepatic HBV DNA analysis, some patients show high levels of liver or serum HBV DNA and a high rate of reactivation (96, 97), whereas, others show low HBV-DNAHBV levels of reactivation rates (93, 98, 99). However, it is postulated that most (if not all) researchers believe that OHB can be found in most recipients of livers from HBsAg-negative and anti-HBc-positive liver donors. Studies have indicated that HBV DNA remains detectable in the serum or PBMCs of patients who are anti-HBV-positive HBV DNA positive and and HBsAg- negative for several years after removal of the HBV-infected liver (99), and extra-hepatic reservoirs serving as the source of reinfection (96). The majority of cases in these studies indicate that the tapering or withdrawal of immunosuppression occurred before reverse seroconversion (100). The likelihood of HBV reactivation would be minimized enormously by immunization and preemptive treatment with nucleos (t)ide analogue drugs as a standard strategy to prevent hepatitis B (79, 101). The need for antiviral therapy in anti-HBcAg- positive and HBsAg- negative patients is not defined well in the literature, and the results of some studies do not support a general recommendation for preemptive use of antiviral therapy in such patients (93, 99). On the other hand, other empirical findings suggest that continued treatment for at least 10 years using an antiviral agent having a high resistance barrier with little or no resistance is required to achieve viral clearance while avoiding reactivation (96, 102). However, close monitoring of HBV DNA levels appears to be warranted. 

## 4. Conclusion

Additional studies in larger cohorts of patients with long-term clinical and laboratory follow-up are warranted to understand the biological basis and significance of occult HBV infection better, and to clarify the possible role exerted by this cryptic infection on the outcome of liver disease and hepatocellular carcinoma development. Individuals with occult HBV infection need to be monitored by follow-up studies to assess the markers of liver damage. Anti-HBc only subjects should be carefully monitored by follow-up studies to assess the significance in various clinical settings. Mass immunization of hepatitis B vaccine is important to control the transmission of HBV. Occult HBV infection is a frequent finding in cases with HIV/HCV co-infection making the virological scenario characterizing this category of patients even more complex than believed formerly. It can be expected that while the burden of chronic hepatitis B will decrease due to the introduction of more effective antiviral therapies, occult HBV infection could become a main concern.
